# Building a healthy mouse model ecosystem to interrogate cancer biology

**DOI:** 10.1242/dmm.049795

**Published:** 2022-09-13

**Authors:** Ryan Devlin, Ed Roberts

**Affiliations:** 1 Beatson Institute for Cancer Research, University of Glasgow, Glasgow G61 1BD, UK; 2 Institute of Cancer Sciences, University of Glasgow, Glasgow G61 1QH, UK

## Abstract

In a recent study, Sargent et al. characterise several novel *Rag1*^−/−^ mouse strains and demonstrate that genetic background strongly influences xenograft development and phenotype. Here, we discuss this work within the broader context of cancer mouse modelling. We argue that new technologies will enable insights into how specific models align with human disease states and that this knowledge can be used to develop a diverse ecosystem of complementary mouse models of cancer. By utilising these diverse, well-characterised models to provide multiple perspectives on specific cancers, it should be possible to reduce the inappropriate attrition of sound hypotheses while protecting against false positives. Furthermore, careful re-introduction of biological variation, be that through outbred populations, environmental diversity or including animals of both sexes, can ensure that results are more broadly applicable and are less impacted by particular traits of homogeneous experimental populations. Thus, careful characterisation and judicious use of an array of mouse models provides an opportunity to address some of the issues surrounding both the reproducibility and translatability crises often referenced in pre-clinical cancer research.

## Abstract (precis)

**Summary:** Diverse and complementary mouse models of cancer enable robust disease positioning and experimental design. Here, we argue that variation in mouse models is central to improving translatability of pre-clinical research.

## Introduction

To unravel complexity, we rely on models recapitulating key features of disease under investigation, and mice have long provided a reliable model to investigate cancer. As early as 1910, Abbie Lathrop developed mouse strains predisposed to developing tumours that, together with work from Leo Loeb, led to the observation that ovarectomy reduced the risk of mammary tumour development (Steensma et al., 2010). By reducing genetic variation, inbred mouse lines not only produced interesting stable phenotypes allowing comparisons across laboratories, but also less variable experimental systems, increasing experimental power (Beck et al., 2000; Festing, 2010; Tuttle et al., 2018). In modern experimental design, there have been further attempts to reduce unwanted variability with environmental standardisation and single-sex protocols, although the benefits of these are controversial (Beery, 2018; Dobson et al., 2019). This reductionist methodology has been, and continues to be, extremely fruitful in evaluating the effects of, for example, specific human-relevant mutations (Campbell et al., 2018; Neidler et al., 2019), exposure to carcinogens (Tsutsui, 1918; Cook et al., 1932; Kennaway, 1955) and potential therapeutic interventions (Muliaditan et al., 2018; Leslie et al., 2022). Indeed, we now have a diverse array of mouse models representing a range of human tumour types, including orthotopic models, genetically engineered models and patient-derived xenografts (Gengenbacher et al., 2017). With this proliferation of model systems, new challenges have arisen, including the need to meaningfully align model species or systems with the human disease states they best represent (Dow et al., 2018) and maximise model translation to human disease and, ultimately, the clinic (Seyhan, 2019). Furthermore, disease models need to be robust enough to ensure that both false positives, which contribute to the crises in reproducibility and translatability, and false negatives, which discard potentially useful therapies, are minimised (Day et al., 2015). Achieving these aims would require a healthy, diverse ecosystem of mouse models to increase the robustness and translatability of data. To paraphrase the statistician George Box, “All models are wrong, but some are useful”, and so rather than aiming to refine an imaginary perfect model, a diverse array of models with complementary strengths and weaknesses within the cancer sciences ecosystem will produce better outcomes in the aggregate (Box, 1976). Several scientific fields have addressed this concept, but a particularly tangible analogy can be found in the field of accident prevention in risk management, known as the Swiss cheese model. In this model, no individual prevention strategy can avert all accidents, but, by layering different systems, each with their own holes, it becomes less likely that an experimental accident will occur, such as an ineffective or unsafe therapy reaching clinical trial stages (Reason, 1990). This Perspective will use this framework to evaluate recent achievements, as well as present what we consider is still needed to generate a robust ecosystem of mouse models of cancer.

“Rather than aiming to refine an imaginary perfect model, a diverse array of models with complementary strengths and weaknesses within the cancer sciences ecosystem will produce better outcomes in the aggregate.”

### The right tool for the right job

**Fig. 1.**
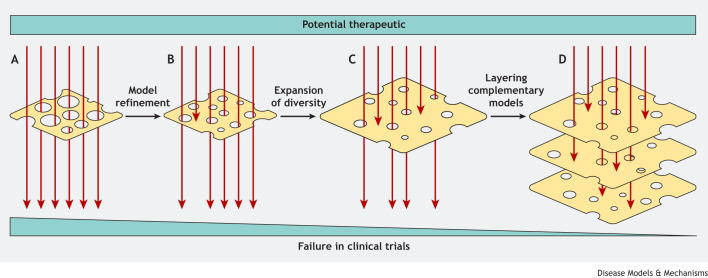
**A visual representation of the Swiss cheese model of accident prevention, modified to represent the strategies for limiting experimental errors and failure in clinical trials.** The limitations of models, represented by the holes, allow experimental errors, represented by red arrows, to occur. (A) In the first scenario, the unmodified experimental model has the greatest experimental accident rate. (B) Model refinement reduces the size of the holes but cannot remove them entirely due to the inherently imperfect nature of models and so errors still can occur. (C) Expanding the size of the layers, through increased intra-model diversity, prevents further errors. (D) Finally, by layering multiple models each with different limitations (different patterns of holes), these errors are minimised. Although one error may occur with one model, it can be caught by the strengths of another.

A diverse array of mouse models, from patient-derived xenografts to genetically engineered mouse (GEM) models with patient-relevant mutations, are now available. In many ways, the current challenge is how to appropriately position these models, that is, to determine which disease states each model most closely aligns with and the areas in which each can most meaningfully be deployed (Dow et al., 2018; Hollern et al., 2018). This change in approach, from continually refining the ‘best’ model to building a suite of more completely characterised models with their own strengths and limitations, has become more common with the realisation that GEM models and patient-derived xenografts have complementary strengths (Richmond and Yingjun, 2008). Although this has been traditionally carried out by studying the histological features of mouse models compared to human pathological specimens (Hollern et al., 2018), a study by Dow et al. compared the genomic and transcriptional landscapes of four mouse models of hepatocellular carcinoma (HCC) and found that some models more faithfully recapitulated different stages and subtypes of human HCC (Dow et al., 2018). Extending this work to more systematically characterise the cancer models currently in use will allow the refinement of the models being used as well as our understanding of the relative utility of each model for specific questions. To return to the analogy of the Swiss cheese model, although refinement of models reduces the size of the holes, better understanding of the limitations of each model can ensure that we do not accidentally utilise multiple interconnected models, leading to unintentional alignment of holes. By combining refinement and increased understanding of the relative strengths and weaknesses, appropriate models can be included in a robust experimental ecosystem (Fig. 1).

“The current challenge is how to appropriately position these models, that is, to determine which disease states each model most closely aligns with.”

**Figure:**
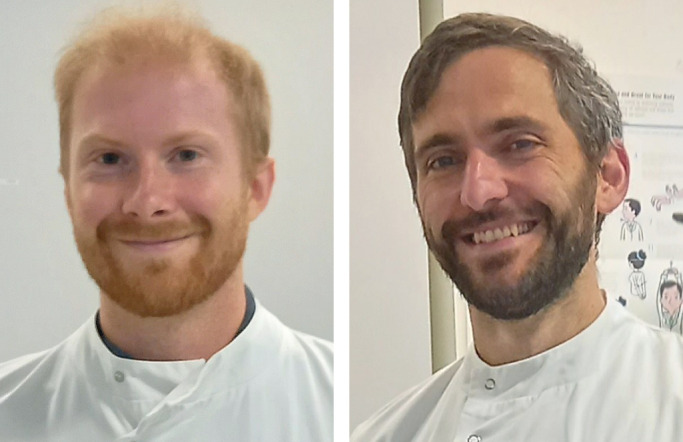
Ryan Devlin (left) and Ed Roberts (right)

### Modelling diverse populations

Ensuring that models are complementary also pertains to the need to ensure that they address the inherent heterogeneities in human cancer at all levels, from the patient population (Jing et al., 2014) through the microenvironment (Binnewies et al., 2018) to clonal cancer cell populations (Reeves et al., 2018). The National Institutes of Health (NIH) and Medical Research Council (MRC) have recognised the need to include genotype and sex variation in experiments to ensure the applicability of research to the highly diverse human population (Waltz et al., 2021). Reintroducing genetic and environmental diversity to previously highly controlled strains enables researchers to address new questions. Indeed, by increasing the genetic and environmental diversity in studies, unique traits of particular inbred lines are diluted, and it is possible to avoid pitfalls of over-interpreting results from very homogeneous populations. For example, B6C3F1 mice are relatively resistant to benzene, meaning that original toxicological studies carried out in these mice predicted a high safe exposure limit (Farris et al., 1996). Later work in an outbred strain of mice more closely recapitulated the human responses to benzene and further identified alleles in C57BL/6 mice that rendered them, and the B6C3F1 mice that are derived from C57BL/6, more resistant (French et al., 2015). In terms of environmental heterogeneity, studies have demonstrated that the immune system of specific pathogen-free mice is more similar to that of human neonates than that of adults (Beura et al., 2016), and that exposure to wild or pet-shop mice could more closely align mice with adult humans (Beura et al., 2016; Rosshart et al., 2019). Unfortunately, this kind of heterogeneity can reduce the power of experiments by increasing variability, raising ethical issues about the number of animals used in research (Festing, 2010). Power can be retained, however, through the use of factorial experimental design, where including genetic background as a blocking variable in the subsequent analysis increases genetic diversity without substantially reducing the power of experiments (Karp and Fry, 2021). This strategy also identifies situations where genetic background may be relevant to observed phenotypes (Karp and Fry, 2021), like the case of benzene sensitivity discussed above. However, it is not always the case that increasing genetic diversity leads to increased phenotypic variation. Several groups have demonstrated that many phenotypic readouts were equally variable within inbred or outbred strains, meaning that an expansion of experiments in these outbred strains could be feasible (Jensen et al., 2016; Tuttle et al., 2018). In our analogy, the use of these diverse populations ensures that we are not discarding or promoting ideas inappropriately because of an over-reliance on near-identical models, which produce pitfalls due to inter-related weaknesses (Fig. 1).

### Opportunities presented by reintroducing diversity

Outbred strains, especially those derived from controlled inbred lines, raise further experimental opportunities, such as allowing quantitative trait locus studies to be carried out in mice (Wei et al., 2020). An example of this approach is the transgenic adenocarcinoma of the mouse prostate cancer model, which was crossed onto an outbred population of mice, and alleles associated with more aggressive tumour development were identified. These alleles were subsequently shown to have similar impact in humans, demonstrating the power of such an approach (Winter et al., 2017). To adapt these approaches to other models of cancer requires a range of genetically diverse mice to be developed that carry any pre-requisite mutations. For example, xenograft studies require the generation of genetically diverse mouse strains lacking adaptive immunity to allow engraftment of human cell lines. In a recent study, Sargent et al. (2022) detail the generation of a suite of *Rag1*^−/−^ mice that do not develop B- or T-lymphocytes, which will allow such factorial experiments or outbred population studies to be carried out in patient-derived xenograft models. Indeed, by producing *Rag1* knockouts in multiple inbred mouse strains, the authors allow such experiments to incorporate 90% of known allelic diversity in the mouse genome. These models benefit from their direct representation of tumour samples from patients (Sargent et al., 2022), which is an approach that has previously been demonstrated to predict therapeutic effects (Woo et al., 2021). Here, in models of breast cancer, leukaemia and glioma, Sargent et al. demonstrate that genetic background significantly impacts the phenotype of the xenograft, highlighting the importance of incorporating genetic diversity when using xenograft models. In this way, it is possible to make mouse experiments more similar to clinical trials or to use factorial experimental design to identify when genetic background is a directly relevant variable, rather than extrapolating findings from experiments using unusually large groups of identical siblings. To return to the Swiss cheese model, this may not reduce the size of the holes, or provide extra layers, but increasing genetic diversity in models expands each layer so that there is less opportunity for potentially good or bad ideas to fall around the sides, leading to inappropriate conclusions.

“Other fields, such as artificial intelligence and ecology, further highlight the strength of redundancy and complementarity within systems.”

## Conclusion

There are lessons to be learned from how other fields address the robustness and reliability of model systems. Although we have focused on a popular model used in accident prevention, other fields, such as artificial intelligence and ecology, further highlight the strength of redundancy and complementarity within systems. By incorporating an appreciation for these principles into cancer research, it should be possible to not only more appropriately utilise existing models, but also encourage a healthy ecosystem of models and experimental design, which will likely increase the relevance of pre-clinical animal research in cancer biology and impact translation to the clinic.
